# Nontuberculous Mycobacteria in Cystic Fibrosis

**DOI:** 10.1007/s40506-016-0092-6

**Published:** 2016-10-22

**Authors:** Kate Skolnik, Gordon Kirkpatrick, Bradley S. Quon

**Affiliations:** 1 Department of Medicine, Division of Respirology, University of Calgary, Calgary, Alberta Canada; 2 Department of Medicine, Division of Respirology, University of British Columbia, Vancouver, BC Canada; 3 Rockyview General Hospital Respirology Offices, 7007 14th Street SW, Calgary, AB T2V 1P9 Canada; 4 Centre for Heart Lung Innovation, University of British Columbia, Vancouver, BC Canada; 5 St. Paul’s Hospital, 8B Providence Wing, 1081 Burrard Street, Vancouver, BC V6Z 1Y6 Canada

**Keywords:** Nontuberculous mycobacteria, Cystic fibrosis, *Mycobacterium avium* complex, *Mycobacterium abscessus*

## Abstract

Nontuberculous mycobacteria (NTM) are found in approximately 10 % of cystic fibrosis (CF) patients, but only a portion will develop NTM disease. The management of CF lung disease should be optimized, including antibiotic therapy targeted to the individual’s usual airway bacteria, prior to considering treatment for NTM lung disease. Those who meet criteria for NTM lung disease may not necessarily require treatment and could be monitored expectantly if symptoms and radiographic findings are minimal. However, the presence of *Mycobacterium abscessus* complex (MABSC), severe lung disease, and/or anticipated lung transplant should prompt NTM therapy initiation. For CF patients with *Mycobacterium avium* complex (MAC), recommended treatment includes triple antibiotic therapy with a macrolide, rifampin, and ethambutol. Azithromycin is generally our preferred macrolide in CF as it is better tolerated and has fewer drug-drug interactions. MABSC treatment is more complex and requires an induction phase (oral macrolide and two IV agents including amikacin) as well as a maintenance phase (nebulized amikacin and two to three oral antibiotics including a macrolide). The induction phase may range from one to three months (depending on infection severity, treatment response, and medication tolerability). For both MAC and MABSC, treatment duration is extended 1-year post-culture conversion. However, in patients who do not achieve culture negative status but tolerate therapy, we consider ongoing treatment for mycobacterial suppression and prevention of disease progression.

## Introduction

Cystic fibrosis (CF) is the most common potentially lethal autosomal recessive disease in Caucasians, found in approximately one in 3000 individuals [1]. While the incidence of CF is lower in other groups, it is known to affect multiple ethnicities [1, 2]. CF results from a mutation in the cystic fibrosis transmembrane conductance regulator (*CFTR*) gene, which encodes a cell-surface chloride ion channel [3]. Reduced CFTR activity leads to impaired chloride ion secretion and viscous secretions, which can block ducts and lumens in various organs [3]. CF is a systemic disease primarily characterized by involvement of respiratory, gastrointestinal, and the male reproductive tracts [3]. However, most of the morbidity and mortality arises from CF lung disease [4, 5]. In the setting of CF, thick airway secretions lead to impaired mucociliary clearance, which predisposes to bacterial colonization and infection [6]. Recurrent cycles of airway infections and chronic inflammation eventually lead to permanent lung damage known as bronchiectasis.

Given the significant burden of pulmonary infection, a critical aspect of CF management revolves around understanding the microbiology of CF lungs in order to prevent and treat infections effectively. The CF lung microbiome is diverse and the spectrum of microbes isolated from CF lungs has increased in the last couple of decades, partly due to laboratory advances [6, 7]. Among these, nontuberculous mycobacteria (NTM) have emerged as noteworthy pathogens in CF that warrant special attention.

## NTM epidemiology

### NTM prevalence and incidence in CF

Reported NTM prevalence in CF ranges from 3 [8•] to 23 % [9]; all studies defined this as a minimum of one positive respiratory culture, but the studies are limited by their retrospective design [8•, 9–19]. The largest studies with 3805, 10,527, and 13,593 patients reported an NTM prevalence of 5 [10], 20 [11], and 3 % [8•], respectively (Table1). Overall, the estimated NTM prevalence in CF is approximately 9 % [8•, 9–19].

Actual NTM disease prevalence, as defined by the American Thoracic Society (ATS) criteria [20], is somewhat unclear. Reported NTM disease prevalence in CF ranges from 4 [12] to 14 % [14] (Table 1). However, this may not be a true representation, as studies differed in NTM disease definition (i.e., some used the 1997 ATS criteria which required three positive respiratory cultures) [21] and none considered radiographic findings.

**Table 1:** Prevalence of NTM positive cultures and NTM lung disease in studies performed since the year 2000

Country	Year	*n*	Prevalence of a single (+) NTM culture % (n)	Prevalence of NTM lung disease* % (n)	Study reference
France	2000–2001	385	8 (31)	-	19
Israel	2000–2003	186	23 (42)	-	9
USA	2000–2007	829	20 (166)	14 (38)^a^	14
Scandinavia	2000–2012	1411	11 (157)	9 (125)^b^	17
USA	2003–2004	986	13 (128)	4 (38)^c^	16
France	2004–2005	1582	7 (104)	4 (57)^d^	12
USA	2011–2012	5403	4 (191)	-	15
USA	2010–2011	10527	14 (1384)	-	11
United Kingdom	2013–2014	3805	5 (190)	-	10
Europe	2015–2016	13593	3 (374)	-	8

*Studies used microbiologic criteria alone to define NTM lung disease

^a^NTM lung disease definition was based on 3 or more positive cultures out of 271 individuals with longitudinal data

^b^NTM lung disease definition was based on 2 or more positive cultures out of all 1411 individuals tested for NTM

^c^NTM lung disease definition was based on 2 or more positive cultures out of all 986 individuals tested for NTM

^d^NTM lung disease definition was based on 2 or more positive cultures out of all 1582 individuals tested for NTM

Geographic variation in NTM prevalence in CF exists within and between countries [10, 12, 16]. This may be due to differences in screening, laboratory techniques, or local NTM subtype prevalence in the environment [16, 22]. NTM incidence in CF appears to be rising in some centers [14, 17]; hypotheses for this include greater surveillance, better diagnostic techniques, and/or shifts in the lung microbiome due to more widespread antibiotic use.

### NTM subtypes

Of the over 150 identified NTM species, only a few have been reported to cause pulmonary disease [20]. The majority (95 %) of NTM isolated from CF patients are *Mycobacterium avium* complex (MAC) (*M. intracellulare* and four *M. avium* subspecies) and *M. abscessus* complex (MABSC) (subspecies *abscessus*, *massiliense*, and *bolletii)* [9, 12, 16, 19]. MAC is generally the most common (representing 75 % of NTM infections in CF), with MABSC accounting for most of the remainder [16]. *M. abscessus* ssp*. abscessus* and *M. abscessus* ssp*. massiliense* comprise the majority of MABSC in CF [23]. Less commonly isolated species in CF are *M. kansasii*, *M. fortuitum*, and *M. gordonae*, among others [9, 12]. Multiple NTM species may be present simultaneously or over time [12, 14, 17].

**Fig. 1:**
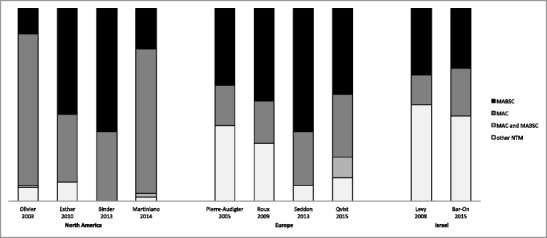
Prevalence of NTM subtypes in CF from different regions of the world.

The proportion of NTM subtypes within a CF population appears to have geographic variation, with MABSC and non-MAC species appearing more common in Europe and Israel (Fig. 1) [9, 10, 15, 16, 24]. Furthermore, there has been an increase in MABSC reported in several centers [12, 16, 19], possibly due to improvements in MABSC identification techniques (16S ribosomal RNA is better at distinguishing MAC from MABSC), increased environmental MABSC, and/or patient-to-patient transmission (although uncommon) [25, 26, 27•].

### Transmission

It was previously believed that NTM pulmonary infection developed exclusively in individuals following environmental acquisition, as mycobacteria, is ubiquitous in soil and water [28]. However, an outbreak of *M. abscesses* ssp *massielense* was first documented at a CF clinic in Seattle, Washington, involving five patients. The index case was smear positive and had recently transitioned from another CF center; isolates from the four outbreak cases were genetically identical and there was overlap with patient visits [25]. Subsequently, there have been reports of NTM spread in England and Hawaii [25, 26, 27•]. The largest outbreak occurred in England, where 11 of 31 patients with MABSC had a shared strain of *M. abscessus* ssp *massiliense* with similar antibiotic resistance patterns despite a lack of exposure to the same antibiotics [26]. Interestingly, two of these outbreaks involved *M. abscessus* ssp*. massiliense* [25, 26]; although other NTM strains are also likely transmissible between humans, this has not been documented in the CF literature to date. The potential for direct or indirect human-to-human transmission underscores the importance of following strict infection control protocols for prevention [29].

## Clinical outcomes of NTM in CF

In the CF population, NTM impact on health outcomes is variable. While MAC and MABSC are the most frequently isolated NTMs in CF, they are also the most clinically significant [9, 12, 15, 16, 30]. However, NTM growth can be transient, and even when persistent, active disease may not develop.

MABSC is more likely to cause invasive NTM lung disease (50 to 80 % of those with positive sputum) compared to MAC (less than half of those with positive sputum) [9, 12, 16, 17, 31, 32].

MABSC also accelerates lung function decline in CF patients compared to uninfected CF controls [14, 32, 33]. In CF, a common measure of lung disease severity is percent-predicted forced expiratory volume in one second (FEV_1_), with lower values indicating more advanced lung disease. In one study, those with MABSC had a greater rate of FEV_1_ % predicted decline than other organisms, including *Pseudomonas aeruginosa* and *Burkholderia cepacia* [33]. However, no lung function decline was noted in patients growing MAC [14]. Similarly, a large study found no significant effect of NTM infection on lung function decline, although analyses were not based on different NTM subtypes [16]. Lastly, CF patients with MABSC are more likely to require transplant or die despite adequate treatment as reported in a recent study [33].

While some NTM play a role in lung disease progression over time, it does not appear to be through more frequent CF pulmonary exacerbations (PEx) [34, 35]. It remains unclear if NTM is merely a marker of worsening lung disease or a causative factor for lung function decline. However, NTM clearance with treatment has been shown to slow the rate of lung function decline [33].

NTM impact on lung transplant outcomes in CF also varies [36]. Generally, those with pre-transplant MABSC are more likely to develop post-transplant invasive MABSC disease, particularly if they are smear-positive [37–40]. Post-transplant MABSC causes significant morbidity, but deaths attributable to MABSC are relatively uncommon in CF and non-CF lung transplant recipients [37–41]. In contrast, post-transplant MAC lung disease is much less frequent, generally lung limited, and has high cure rates [37, 38]. All CF patients should be screened for NTM pre-transplant and, if present, started on treatment prior to transplant listing [42]. Progressive pulmonary or extra-pulmonary NTM disease despite appropriate therapy or treatment intolerance is considered a transplant contraindication [42].

## NTM risk factors

Within the CF population, multiple potential risk factors for NTM pulmonary infection have been investigated; while some are consistent between studies, the majority of factors demonstrate inconsistent associations. Clinical characteristics, including baseline lung function, have generally not been linked to NTM risk in CF [8•, 15–17, 43]. The most consistently identified NTM association is *Aspergillus fumigatus* co-infection [14, 16, 24, 31, 44]. Those with allergic bronchopulmonary aspergillosis (ABPA) also had higher rates of NTM in many large studies [8•, 15, 18, 24], but not in all [45, 46]. Other microorganisms do not appear to be associated with increased NTM risk [8•, 9, 14, 15, 24]. Younger age, pancreatic insufficiency, and lower baseline lung function are more common with MABSC compared to MAC in some studies [12, 14, 15, 30, 32, 46].

With increased macrolide use to reduce CF PEx and the parallel increase in NTM prevalence, there is concern that this practice could predispose to active NTM pulmonary disease. A proposed mechanism is macrolide-mediated impairment in macrophage function, leading to reduced intracellular bacterial killing [46]. However, results from existing evidence are mixed with some studies showing an increased risk of NTM [9, 45], and more recent studies demonstrating no or decreased NTM risk with chronic macrolide use [8•, 15, 31, 47]. The influence of macrolides on NTM risk is inconclusive, and macrolides should still be considered if indicated. Regarding other medications, a recent study found that several inhaled medications were associated with increased NTM risk [8•]. This has not been validated in other studies nor investigated prospectively. Interestingly, neither inhaled nor oral corticosteroids were associated with a higher likelihood of NTM infection in several large studies [8•, 17, 18, 43, 46].

## NTM diagnosis

### NTM culture identification

When clinical suspicion exists, patients should collect expectorated sputum samples on three consecutive mornings to increase yield [20, 48•]. Induced sputum samples may be obtained in subjects unable to readily produce sputum [20, 48•]. When both expectorated/induced sputum samples are non-diagnostic or negative but a high index of suspicion for NTM lung disease remains, bronchoscopy should be pursued for bronchoalveolar lavage [20, 48•]. Transbronchial biopsies are not routinely recommended in CF patients and should particularly be avoided in those with severe bronchiectatic changes due to higher pneumothorax risk [48•]. Cough swabs, induced sputum samples, and bronchoscopy are not required as part of routine surveillance in individuals unable to expectorate spontaneously [48•]. Routine NTM surveillance should be performed at least annually [48•].

Specimens should be processed within 24 h and require decontamination to prevent other bacterial overgrowth and to facilitate NTM detection [20]. Additional decontamination is needed if Gram-negative bacteria persist, a more prevalent problem in CF [48•, 49]. Respiratory specimens should be incubated on both liquid media (which improves mycobacterial yield) and solid media (which allows for growth rate quantification and detection of multiple simultaneous NTM strains) [20].

Due to varying clinical implications, all NTM isolates should be identified at the species level (with the exception of MAC) and the sub-species level in the case of MABSC [20, 48•]. Initial mycobacterial identification may occur by acid-fast bacilli detection with Ziehl-Neelsen stain (but sensitivity varies based on infectious burden). NTM cultures can take anywhere from 7 days (as with MABSC, *M. fortuitum*, and *M. chelonae*) to 12 weeks to grow [20].

Standard laboratory practice for NTM speciation entails either high-performance liquid chromatography (HPLC) or molecular methods, which are generally preferred [20, 48•, 50, 51]. Molecular NTM genotyping options include commercially available DNA probes, PCR product-restriction enzyme analysis (PRA), or 16S ribosomal RNA sequencing [20, 48•]. Commercially available NTM-specific DNA probe assays and PRA testing have high sensitivity and specificity [20, 48•], but the former is costly and only available for a few NTM types and the latter is not widely available [20]. NTM-specific 16S ribosomal RNA sequencing has high sensitivity, superior specificity, provides better discrimination among rapid growers, and is widely available [20, 48•]. MABSC sub-speciation is done by multi-locus sequence typing of *hsp65*, *rpoB*, and *secA* genes, a recently validated technique with fair accuracy [52, 53]. Antibiotic susceptibility profiles can provide a clue to the MABSC subspecies (i.e., macrolide sensitivity is suggestive of *M. abscessus* ssp. *massiliense*); however, confirmatory testing is required [48•].

### NTM lung disease

A diagnosis of NTM pulmonary disease must meet both clinical and microbiologic criteria as outlined by ATS guidelines, with exclusion of other etiologies [20]. Clinical criteria include persistent pulmonary symptoms and radiographic changes (nodular and/or cavitary opacities on radiograph or bronchiectasis with nodules on CT scan), and microbiologic criteria require persistent growth of NTM in respiratory specimens [20]. However, this definition is problematic in CF and poses a challenge in the accurate diagnosis of NTM lung disease. Individuals with CF tend to have chronic productive cough, shortness of breath, and weight loss which can fluctuate and progress, making it difficult to distinguish the natural course of CF from evolving NTM lung disease. Furthermore, CF is characterized by bronchiectatic changes, nodularity from mucus plugging, and has a predilection for the upper and mid lung zones. Consequently, using the standard diagnostic criteria for NTM lung disease can lead to over-diagnosis of disease in CF, and potentially, over-treatment and unnecessary exposure to antibiotics. Unfortunately, there are no CF-specific criteria for the diagnosis of NTM lung disease that would address these potential issues.

CF clinicians must use their judgment to decide if the above-mentioned respiratory symptoms and radiographic features have progressed from baseline and are due to NTM [48•]. A key part of this process entails reviewing the patient’s overall CF disease status and optimizing management of CF lung disease and comorbidities that could affect respiratory status (including malnutrition, CF-related diabetes, and asthma) [48•]. It is generally recommended to treat PEx caused by more typical pathogens (e.g., *Pseudomonas* spp.) to see if the patient returns to baseline, prior to proceeding with more involved diagnostic tests and treatments for NTM [48•].

## NTM treatment and outcomes

### Treatment indications

Individuals who are NTM culture positive but who do not meet ATS criteria for disease should be followed closely [48•]. Everyone meeting criteria for NTM lung disease should be considered for therapy; however, treatment decisions should be individualized [48•, 54]. It may be reasonable to monitor individuals with mild CF lung disease, MAC lung disease with mild symptoms and radiographic changes, or a high probably of drug intolerance or drug interactions [54]. Conversely, CF patients with MABSC and/or severe CF lung disease should generally be treated in the absence of contraindications [54].

### Treatment challenges

NTM lung disease treatment is challenging due to long, complicated, and difficult to tolerate antibiotic regimens [54, 55]. Furthermore, in vitro antibiotic susceptibility and in vivo response may not correlate [54, 56, 57]. This may be partly due to NTM biofilm formation, which has only more recently been recognized in the lungs and appears to be limited to a few species (including MAC and MABSC) [57, 58]. Treating NTM in CF is associated with additional difficulties. Achieving therapeutic antibiotic concentrations is a challenge due to the higher volume of distribution of antibiotics, increased renal clearance, and possibly decreased gastrointestinal absorption [54, 56]. Increased macrolide exposure in CF patients may lead to greater macrolide resistance [53]. Lastly, treatment tolerance and efficacy may be influenced by interactions with other chronic medications.

### MAC treatment

Standard MAC treatment consists of a macrolide (azithromycin 250 mg or clarithromycin 1000 mg once daily), rifampin 10 mg/kg daily up to 600 mg, and ethambutol 15 mg/kg daily [20, 48•]. Treatment duration is for 1 year following culture conversion (when respiratory specimen culture first becomes negative) [20, 48•]. It is important to note that the culture conversion must be sustained on serial repeats and should there be even one positive culture, the clock would need to be reset on treatment duration [20, 48•]. Azithromycin is generally the preferred macrolide (despite there being a lack of evidence to suggest that it is superior to clarithromycin), since it is better tolerated, has fewer drug-drug interactions, and has the added benefit of reducing CF PEx through an anti-inflammatory mechanism [20, 48•]. Based on ATS guidelines, intermittent dosing is considered acceptable in nodular NTM lung disease, but it is not advised in patients with CF (regardless of severity) as intermittent dosing is less likely to achieve therapeutic drug levels in this group [20, 48•]. A parenteral aminoglycoside should be strongly considered in those with severe cavitary disease to improve treatment outcomes, and new CF-specific guidelines suggest that smear-positive MAC and/or cases associated with systemic signs of illness are also indications for parenteral aminoglycoside use [48•, 59]. Generally, intravenous (IV) amikacin is the drug of choice; however, intramuscular streptomycin is a reasonable alternative with good efficacy against MAC [60]. When used for MAC treatment, amikacin or streptomycin should be administered for one to three months [48•]. Dosing of these aminoglycosides three times a week appears comparable to daily lower dose administration without increasing toxicity [49, 61].

Guidelines recommend testing for macrolide resistance in the following situations: all new MAC cultures, when MAC regrows after recent eradication, or when there is treatment failure (defined by some as failure to convert to culture negative after 4 months of adequate therapy) [20, 59]. Of note, there is no additional proven value in susceptibility testing for other antibiotics in the setting of new MAC infection [20].

Despite prolonged courses of multidrug therapy, effective cure for MAC lung disease is just under 60 % in the general population (without immune suppression or CF) [62]. There are no randomized controlled trials comparing NTM treatment regimens in CF; therefore, knowledge regarding drug efficacy is derived from studies in the broader population. Several studies of MAC treatment in non-CF bronchiectasis, which included macrolide, ethambutol, and rifamycin therapy in combination with IV amikacin (or streptomycin), demonstrated culture conversion in approximately 50 % of subjects at 6 to 12 months [63–68]. The average time for culture conversion was 4 months, with a wide range among the studies [63–68]. MAC recurrence rates in the non-CF population are significant despite adequate therapy, reaching 40 % after 3 years of follow-up [20].

Although there are no controlled clinical trials regarding MAC treatment efficacy and recurrence rates in CF, a few case series exist. Macrolide-based triple therapy regimens (some including IV amikacin) lead to 90 % (*n* = 11) and 100 % (*n* = 5) culture conversion rates in two separate studies, but the authors did not report on the timing of conversion or recurrence rates [69, 70]. Although the conversion rates appear much higher in these studies than the non-CF bronchiectasis population, this needs to be interpreted with caution due to the small sample size, milder CF lung disease in most of the cases, and the variability in treatment regimens [69, 70]. Importantly, while some individuals may never be able to achieve cure, ongoing treatment for mycobacterial suppression and prevention of disease progression should still be considered if the therapy is reasonably well tolerated [48•, 59].

### MABSC treatment

MABSC treatment is more complex and challenging than the treatment of other NTM species. Inducible macrolide resistance is especially problematic and common in *M. abscessus* ssp*. abscessus* and *M. abscessus* ssp. *bollettii* infections as they harbor the erythromycin ribosome methyltransferase (*erm41*) gene [71]. The *erm*41 gene, when induced by macrolide exposure, leads to ribosome methylation, which results in macrolide resistance [71]. *M. abscessus* ssp*. massiliense* has a partial *erm* gene deletion, which prevents resistance by this mechanism [48•, 53]. However, macrolide resistance can still occur in all MABSC through other, less common mechanisms [28]. Deriving an adequate treatment regimen is challenging as the antibiotic susceptibility profile, patient comorbidities, and intolerances all must be considered.

The therapeutic approach is similar for all MABSC subspecies. As with other NTM, MABSC treatment requires at least three active agents simultaneously, and the regimen should be continued for at least 1-year post-culture conversion. Similar to other NTM, MABSC therapy is guided but not dictated by antibiotic susceptibility [48•]. In contrast to MAC, MABSC treatment is divided into an induction phase (usually one to three months of IV antibiotics) and a maintenance phase (continued until the end of treatment); induction duration is judged by infection severity, treatment response, and medication side effects (but not necessarily culture conversion) [48•]. Furthermore, IV amikacin should always be used for MABSC management in the absence of contraindications [48•]. For the induction phase, a combination of an oral macrolide (azithromycin 250 mg daily preferred), IV amikacin (15 mg/kg/day in divided doses), and at least one other IV agent (cefoxitin 200 mg/kg/day divided in three doses, tigecycline 50 mg once to twice daily, or imipenem 1 g twice daily) is recommended [48•]. Cefoxitin is often a starting point as it is generally accessible and relatively well tolerated [20]. Inducible macrolide resistance is more likely with clarithromycin, providing another reason why azithromycin is preferred for MABSC treatment [72]. For the maintenance phase, the oral macrolide is continued, and parenteral antibiotics are exchanged for nebulized amikacin (250 to 500 mg twice daily) and two to three other oral antibiotics [48•]. Oral agents often used in MABSC treatment include minocycline, linezolid, clofazimine, moxifloxacin and, less often, co-trimoxazole or ethambutol [20, 48•]. It may be difficult to predict which combination the patient will respond to and tolerate and therefore empiric choices are often required, with modification based on initial response and intolerances.

There are no randomized controlled trials of MABSC therapy in the general population or in CF; however, there is MABSC treatment outcome data in non-CF populations from several clinical studies [48•]. In a study of 56 non-CF subjects that compared *M. abscessus* ssp*. massiliense* and ssp. *abscessus* infections and treatment outcomes, all individuals were treated with clarithromycin and a standard 4-week induction period of IV amikacin and cefoxitin followed by clarithromycin, ciprofloxacin, and ethambutol for 24 months (minimum 12 months post-negative culture) [73]. *M. abscessus* ssp. *massiliense* demonstrated a better treatment response with more sustained negative cultures compared to *M. abscessus* ssp*. abscessus* (88 vs 25 %) [73]. Interestingly, there was a high rate of doxycycline and fluoroquinolone resistance while macrolide and amikacin resistance at baseline was low in both groups [73]. In a retrospective observational study of 157 CF patients with NTM (where approximately half had MABSC), MABSC culture conversion occurred in one third and 25 % died, despite treatment; the nature and success of treatment regimens were not discussed [17].

Despite intensive treatment regimens, MABSC culture conversion and cure rates are low in CF [17], and similar to non-CF studies [73]. Consequently, MABSC cure is often not achievable, and while it should still be attempted, guidelines indicate that a more realistic goal is sustained symptom reduction, radiographic response, and/or smear negative status [20].

### Treatment monitoring and side effects

Sputum cultures should be monitored every four to eight weeks until culture conversion and less frequently thereafter [20, 48•, 59]. A CT scan of the chest is advised at the start and end of treatment [48•]. Although not routinely recommended, an interim CT scan may be useful if there is clinical worsening on treatment (not explained by an exacerbation) or if there is a failure of culture conversion (as cavitary disease may have developed).

In addition to repeat culturing and imaging, monitoring should include symptom review and clinical and laboratory assessment for side effects. Medication side effects and recommended monitoring are summarized in the general and CF-specific NTM treatment guidelines [20, 59]. Irrespective of the regimen, monthly bloodwork for cell counts, renal, and hepatic function are advised [20, 59]. Drug level monitoring is required for amikacin but is generally not necessary for most other medications [20, 59].

### Management of treatment failure

Treatment failure refers to an inability to attain culture conversion within a reasonable time frame; many define this as four months; however, this definition may be too strict as some individuals may still respond but take longer to convert their cultures [20, 59]. Regardless, non-responders or slow responders should prompt reassessment of treatment after a few months. Possible reasons include medication non-adherence, antibiotic resistance, suboptimal drug levels (due to under-dosing, increased clearance, or decreased absorption), or suboptimal drug delivery to the active site (due to biofilm formation, viscous mucus, or cavities). In the setting of treatment failure, full antibiotic susceptibility testing should be performed (irrespective of the NTM type). If susceptible, antibiotic doses could be maximized before changing to a different regimen. Drug level monitoring may also be useful if there are concerns about suboptimal medication absorption or increased clearance.

MAC treatment failure has been associated with cavity formation, smear positivity, macrolide resistance (at the start or emerging during treatment), prior MAC treatment, poor lung function, and intolerance to treatment [22, 63–66, 68]. Macrolide resistance was as high as 15 % in one study, but is lower when adequate triple antibiotic therapy is used [20, 48•, 63]. Although there are no CF-specific studies, guidelines suggest using IV amikacin, changing rifampin to rifabutin, and considering clofazimine and/or moxifloxacin for macrolide-resistant MAC [20]. Evidence from the non-CF population also suggests those with intolerance/resistance to rifamycins, have good outcomes with clofazimine, ethambutol, and a macrolide [74]. Finally, in the setting of cavitary or treatment refractory MAC infections, those individuals treated with IV amikacin and localized surgery had the best outcomes in non-CF bronchiectasis [20]; however, lung resection surgery is generally avoided in CF except in extenuating circumstances [48•].

With MABSC, treatment failure is more likely to occur with non-*massiliense* subspecies, macrolide-resistance, lack of surgical intervention, and four or more positive sputum cultures [17, 72, 75, 76]. Possible options for MABSC infection refractory to first-line therapy is exchanging the beta-lactam for IV tigecycline and possibly adding clofazimine [77, 78•]. An in vitro susceptibility study involving 65 MABSC strains suggested that tigecyline and clofazimine had the greatest synergy (in almost half of the isolates) when multiple antibiotic combinations were evaluated [77]. A non-comparative open label clinical treatment trial for rapidly growing NTM (mostly MABSC) that included 21 CF patients was recently published [78•]. All were given IV tigecycline along with macrolides (75 %) [78•]. IV aminoglycoside, linezolid, IV beta-lactam, and fluoroquinolones were used in 75, 60, 40, and 22 %, respectively [78•]. Overall, 75 % of CF subjects had clinical improvement based on symptoms, CT findings, and/or sputum culture conversion [78•]. However, the majority of subjects had nausea/vomiting and many discontinued tigecycline prematurely [78•]. The study found that drug intolerance was more likely with 100 vs 50 mg daily and with loading doses; they recommended that prophylaxis with anti-emetics, avoidance of a loading dose, and slow up-titration to the target dose (starting from 25 mg OD and increasing to 100 mg OD over a few weeks) could help improve drug tolerability [78•]. This study marks one of the first CF-specific studies of NTM treatment and provides evidence for tigecycline efficacy and safety as part of MABSC therapy in this population. As with cavitary or treatment-resistant MAC, those with severe MABSC have been reported to have better outcomes with lung surgery [20, 76], but its role in CF is limited as lung resection surgery is generally avoided [48•].

## New therapies and future research opportunities

New developments in NTM management include identification of several potential therapeutic targets and novel treatments. Gallium compounds appear to impair *M. abscessus* ssp*. abscessus* growth in human macrophages by disrupting iron uptake [79]. MgtC, an *M. abscessus* ssp. *abscessus* virulence factor, is induced upon entering human macrophages; blocking this factor was protective against inhaled MABSC in animal models and may be a novel treatment target [80]. *M. abscessus* ssp. *abscessus* has also been shown to produce BlaMab, a beta-lactamase, which may partly account for suboptimal response despite cefoxitin or imipenem use [81]. Avibactam, a beta-lactamase inhibitor, blocked BlaMab in human macrophages and animal models, suggesting that combining this with beta-lactams may improve MABSC treatment efficacy [81]. Piperinodol, which disrupts mycolic acid transport, has fair anti-MABSC activity in human macrophages and zebrafish models [82].

Inhaled liposomal amikacin for maintenance treatment has also drawn interest. In a randomized placebo controlled trial, CF subjects with NTM lung disease refractory to standard therapy were assigned to 590 mg OD inhaled liposomal amikacin or placebo, in addition to their standard CF treatments and ongoing NTM therapy [75]. The group of 90 patients was stratified based on MAC (64 %) and MABSC (36 %) [83•]. At the end of the 6-month treatment period, there was a statistically significant increase in culture negativity overall and for the MAC group. However, there were no differences in the MABSC group vs placebo (which may have been partly been due to the study being underpowered) [83•]. Although there are no human clinical trials comparing free inhaled amikacin to liposomal amikacin, mouse models showed liposomal amikacin was as effective as a higher concentration of free amikacin against MAC [84]. These findings suggest that liposomal amikacin might be beneficial as an adjunct in the management of difficult to treat MAC (where criteria for IV amikacin are not met or there is a contraindication). This formulation of amikacin was well tolerated and may be useful in MABSC treatment (but needs to be investigated further in this context). Moving forward, NTM treatment clinical trials are urgently needed in CF, particularly the development of more effective regimens for MABSC.

## Abbreviations

CF: Cystic fibrosis
CFTR: Cystic fibrosis transmembrane conductance regulator
CT: Computed tomography
erm41: Erythromycin ribosome methyltransferase
FEV_1_: Forced expiratory volume in one second
IV: Intravenous
MABSC: *Mycobacterium abscessus* complex
MAC: *Mycobacterium avium* complex
PEx: Pulmonary exacerbation
